# Effects of Heat Treatment on the Physicochemical Properties and Electrochemical Behavior of Biochars for Electrocatalyst Support Applications

**DOI:** 10.3390/ma16165571

**Published:** 2023-08-10

**Authors:** Rocío García-Rocha, Sergio M. Durón-Torres, Salvador A. Palomares-Sánchez, Antonio Del Rio-De Santiago, Ivone Rojas-de Soto, Ismailia L. Escalante-García

**Affiliations:** 1 Doctorado Interinstitucional de Ingeniería y Ciencias de Materiales, Universidad Autónoma de San Luis Potosí, Sierra Leona 550, Lomas 2da. Sección, San Luis Potosí 78210, Mexico; rocio.garcia.rocha@gmail.com; 2 Unidad Académica de Ciencias Químicas, Universidad Autónoma de Zacatecas, Campus Siglo XXI, Ed. 6, km. 6 Carr. Zacatecas-Guadalajara, Zacatecas 98160, Mexico; duronm@uaz.edu.mx; 3 Facultad de Ciencias, Universidad Autónoma de San Luis Potosí, Av. Chapultepec 1570, San Luis Potosí 78295, Mexico; sapasa04@fciencias.uaslp.mx; 4 Unidad Académica de Ingeniería Eléctrica, Universidad Autónoma de Zacatecas, Ramón López Velarde 801, Zacatecas 98000, Mexico; risa008537@uaz.edu.mx (A.D.R.-D.S.); ivonerds@uaz.edu.mx (I.R.-d.S.)

**Keywords:** biochar, electrocatalyst supports, carbon, H_2_ and N_2_ atmospheres

## Abstract

The present work reports the synthesis and the physicochemical characterization of biochar from the organic wastes of nopal (*Opuntia Leucotricha*), coffee grounds (*Coffea arabica*) and Ataulfo mango seeds (*Mangifera indica*) as alternative electrocatalyst supports to Vulcan XC-72 carbon black. The biochars were prepared using pyrolysis from organic wastes collected at three temperatures, 600, 750 and 900 °C, under two atmospheres, N_2_ and H_2_. The synthesized biochars were characterized using Raman spectroscopy and scanning electron microscopy (SEM) to obtain insights into their chemical structure and morphological nature, respectively, as a function of temperature and pyrolysis atmosphere. A N_2_ adsorption/desorption technique, two-point conductivity measurements and cyclic voltammetry (CV) were conducted to evaluate the specific surface area (SSA), electrical conductivity and double-layer capacitance, respectively, of all the biochars to estimate their physical properties as a possible alternative carbon support. The results indicated that the mango biochar demonstrated the highest properties among all the biochars, such as an electrical conductivity of 8.3 S/cm^−1^ at 900 °C in N_2_, a specific surface area of 829 m^2^/g at 600 °C in H_2_ and a capacitance of ~300 mF/g at 900 °C in N_2_. The nopal and coffee biochars exhibited excellent specific surface areas, up to 767 m^2^/g at 600 °C in N_2_ and 699 m^2^/g at 750 °C in H_2_, respectively; nonetheless, their electrical conductivity and capacitance were limited. Therefore, the mango biochar at 900 °C in N_2_ was considered a suitable alternative carbon material for electrocatalyst support. Additionally, it was possible to determine that the electrical conductivity and capacitance increased as a function of the pyrolysis temperature, while the specific surface area decreased for some biochars as the pyrolysis temperature increased. Overall, it is possible to conclude that heat treatment at a high temperature of 900 °C enhanced the biochar properties toward electrocatalyst support applications.

## 1. Introduction

Usually, plant residues accumulate in the open air to facilitate their drying, thus reducing their volume. Later, these residues can have three main destinations: incorporation into the soil of crops, burning in situ or simply being left to continue their course of decomposition in landfills [1]. However, each of these options carries a risk, either of air pollution as a result of burning or to health by promoting the appearance of harmful fauna that can cause diseases in humans.

In the search for a direct application of this organic waste, which is economical and friendly to the environment, efforts have been made to develop its use in the production of biofertilizers [2] or biofuels [3]. However, none of these options meets the above requirements since in most cases, the waste must be collected and transported to processing centers, which increases costs; otherwise, the alternative option is to leave the waste in municipal landfills, where the separation of biomass can be conducted for different purposes, but not for the production of energy since there are no facilities for this purpose at the municipal level, so they only accumulate, causing sources of infection and damage to the environment [4,5]. On the other hand, the synthesis of biocarbons is friendly to the environment, in addition to being not only economical, but also providing added value to the organic waste [6] and having extremely versatile applications; for instance, biocarbons can be used for the filtration and adsorption of contaminants [7,8] and as soil rectifiers [9,10], among other applications; therefore, this can be an excellent material alternative to carbon, which deserves further research.

There are little known alternatives for the treatment of organic waste, such as the clean process that results in usable products, among them the so-called “biochar”, a given scientific term. According to Lehmann and Joseph [11], a biochar is defined as a carbon-rich product when biomass such as wood, manure or leaves is heated in a closed container in the absence of oxygen; this process is also known as pyrolysis. The chemical composition of the biomass feedstock and the temperature as well as the atmosphere of the pyrolysis are some of the factors that significantly influence the physical and chemical nature of the biochar [12].

Some of the potential applications of biochars are their use in the production of activated carbon, which can be used as a supercapacitor [13], and in the remediation of heavy-metal-contaminated soils [14], as well as in energy storage systems such as hydrogen fuel cells or batteries [15] and even in microbial fuel cells as cathodes for the oxygen reduction reaction [16]. Since the pyrolysis conditions have a direct effect on the chemical composition of the surface and on the physical properties of the final material (i.e., physical morphology, specific surface area, electrical conductivity, capacitance), they can be arranged in different ways to find new biochar materials with tuned properties for specific applications. For these reasons, biocarbons (biochar) with an enhanced surface area and electrical conductivity can be suitable as electrocatalyst supports of metallic or bimetallic nanoparticles in order to easily facilitate the transport of electrons toward electrochemical reactions of scientific interest [17].

Some studies reported the use of a chemical or physical activation process of the biochar before or after pyrolysis for catalyst support applications [18]. Although an activation process is usually considered to improve the physical parameters of the biochar, such as the specific surface area, there are reports in the literature where some biochars have exhibited specific surface areas of 435 m^2^/g and 687 m^2^/g without any activation treatment; these values are comparable with those of activated biochars [19,20]. Thus, the activation of biochar does not guarantee the obtainment of high-specific-surface-area materials. An enhancement in the specific surface area of biochar has been achieved by modifying only the pyrolysis temperature, as reported elsewhere [21]. Additionally, there are authors who showed that it is possible to use biochars as a catalyst support without the need for an activation process [19,22].

In the present work, biochars were obtained from nopal (*Opuntia Leucotricha*), coffee grounds (*Coffea arabica*) and the seed of mango Ataulfo (*Mangifera indica*) wastes using pyrolysis in a H_2_ atmosphere and a N_2_ atmosphere at three different temperatures (600, 750 and 900 °C). The obtained materials were characterized using Raman spectroscopy in order to obtain insights into the crystallinity and disorder of the materials. Scanning electron microscopy (SEM) was performed to study the morphology of the obtained biochars. Overall, micrometric agglomerates <2 μm were formed with smaller particles on the surface, particularly on the nopal (*Opuntia Leucotricha*) and Ataulfo mango seed (*Mangifera indica*) waste samples. The adsorption–desorption method of N_2_ was employed to evaluate the specific surface area of the biochars. At a pyrolysis temperature of 600 °C, mango Ataulfo (*Mangifera indica*) in H_2_ atmosphere (829 m^2^/g) and nopal (*Opuntia Leucotricha*) in N_2_ atmosphere (766 m^2^/g) demonstrated the highest surface area among the obtained biochars. However, there are materials that, regardless of the temperature, had a similar specific surface area at different pyrolysis temperatures such as nopal (*Opuntia Leucotricha*) biochar in H_2_ atmosphere with ~500 m^2^/g. Conductivity measurement by the dual probe method was carried out at different applied pressures. From these experiments, the most conductive biochars were those prepared from coffee grounds (*Coffea arabica*) in N_2_ atmosphere at 900 °C, ~10 S cm^−1^. Additionally, mango pyrolyzed at 900 °C showed a conductivity similar to the coffee biochar, irrespective of the pyrolysis atmosphere. Finally, cyclic voltammetry was performed to investigate the electrochemical profile of the biochars and to determine their double-layer capacitance. The results showed that some of our biochars have a similar profile to XC-72 Vulcan carbon, which is commonly used as an electrocatalyst support.

## 2. Materials and Methods

### 2.1. Preparation of Biochars

**Figure 1:**
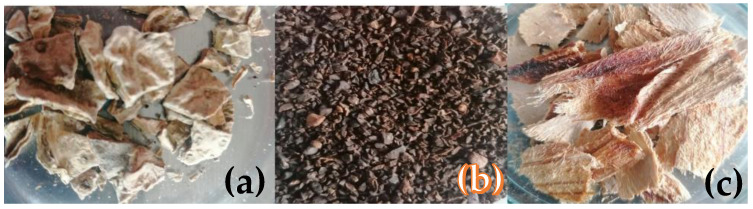
Images of dried biomass samples before the biochar stage: (**a**) nopal, (**b**) coffee and (**c**) mango.

Nopal (*Opuntia Leucotricha*) and Ataulfo mango seed (*Mangifera indica*) wastes were collected in May, and coffee waste (*Coffea arabica*) was obtained directly from the filter of a coffee maker after coffee service. The mango seed was washed with water to remove any remaining fruit while nopal and coffee waste were used as collected. The three organic wastes were dried at room temperature for a week, as shown in Figure 1; then, they were placed in an oven at 80 °C for 24 h before pyrolysis.

The pyrolysis was carried out as reported by Quintana Melgoza et al. [23] as briefly described. Once dried, each of the organic waste was placed in a horizontal tube furnace Barnstead 21,100 (Thermo Fisher Scientific, Waltham, MA, USA) with a flow of 120 cm^3^/s under a H_2_ atmosphere or N_2_ atmosphere at 600 °C, 750 °C and 900 °C for 1 h. Here, the furnace was heated at a ramp temperature of 10 °C/min until the desired temperature was reached. After 1 h, the gas flow was stopped and the pyrolyzed sample was cooled down to room temperature without a cooling ramp [23].

After pyrolysis, the obtained materials were ground in an agate mortar and then in a Fritsch pulverisette 23 ball mill (FRITSCH Milling and Sizing, Inc., Pittsboro, NC, USA) at 30 oscillations/s for 20 min in order to reduce the grain size to the maximum and avoid agglomerates.

### 2.2. Physicochemical Characterization

The structural analysis of the obtained materials was carried out in a Raman XploRA Syncerity Spectrometer (Horiba, Kyoto, Japan) with a 532 nm laser, and an acquisition time of 15 qs in a range from 100 to 3000 nm. The morphology of the wastes after pyrolysis was analyzed by scanning electron microscopy (SEM), using a Jeol JSM-6510lV brand microscope (JEOL, Akishima, Japan) with 3 nm nominal resolution and 30 kV voltage. The specific surface area was determined using nitrogen adsorption/desorption at −196 °C (77 K) in a ChemiSorb 2720 Instrument (Micromeritics, Norcross, GA, USA), through the ChemiSoft TPx software. The samples were then degassed with N_2_ at 120 °C for 30 min prior to measurements.

**Figure 2:**
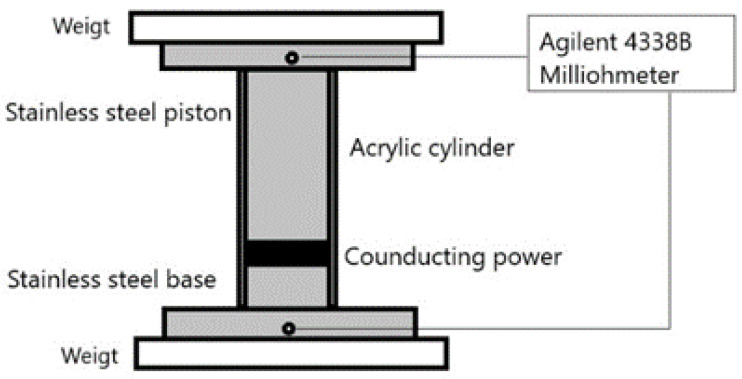
Schematic representation of conductivity cell for 2-point measurement.

The electrical conductivity of the biochar samples was evaluated by a 2-point method using a conductivity cell designed and built in a laboratory. This cell was similar to that reported in [24,25]. The conductivity cell was of a cylindrical shape with a stainless steel piston 7.6 mm in diameter and that was moved in the y-axis direction along a transparent acrylic tube, in which the prepared material was placed. In addition, the acrylic tube was marked with a mm scale to measure the length of the compacted powder at a desired pressure. The lower end of the cylinder was sealed by a stainless steel base. Finally, a stainless steel plate was attached to the end of the metal barrels that were connected to an Agilent 4338B milliohmmeter (Agilent Technologies, Santa Clara, CA, USA). A schematic representation of the conductivity cell is depicted in Figure 2. Once the sample was placed in the conductivity cell, a Carver Model 4386 hydraulic press (Carver Inc., Wabash, IN, USA) was used to compress the studied material at different pressures from 62 kPa to 620 kPa. The biochar materials were compressed to obtain a 1 mm thick pellet at 620 kPa with a cross-sectional area corresponding to the diameter of the acrylic tube in order to obtain comparable and reproducible data [26]. Additionally, the conductivity measurements were performed three times for each biochar to test the reproducibility. The average of the three recorded values at 620 kPa is reported in Section 3.

Then, the electrical conductivity of the pyrolyzed materials was calculated indirectly, first by calculating the resistivity, using Equation (1):

(1) $$\rho_{z}=\frac{R_{m}.\;A}{L}$$

where ρ_z_ is the resistivity of the material in Ω⋅m, R_m_ is the resistance in Ω, L is the thickness of the compressed material, and A is the cross-sectional area of compressed material.

Finally, the electrical conductivity of the prepared biochar was calculated by Equation (2):

(2) $$\sigma =\frac{1}{\rho_{z}}$$

It has been widely reported that the capacitance, electrical conductivity and specific surface area are among the most important physical properties for designing a carbon-based electrocatalyst support. This is due to the fact that a larger specific surface area allows a greater amount of charge to be deposited on the surface, resulting in a high ratio of active area per volume unit, which favors the diffusion of reactants and products as well as the electron transference [27].

### 2.3. Electrochemical Characterization

Electrochemical studies of the prepared carbon materials were performed by cyclic voltammetry (CV) in a conventional three-electrode electrochemical cell using a potentiostat/galvanostat VersaStat 3 (AMETEK SI, Oak Ridge, TN, USA). First, an ink was prepared by adding 6 mg of the biocarbon (biochar) of nopal (Opuntia Leucotricha), coffee (Coffea arabica) or Ataulfo mango (Mangifera indica) and 6 µL of 10 wt.% Nafion^®^ (Aldrich, Burlington, MA, USA)solution in 20 µL of ethanol. Both reagents were purchased from Sigma-Aldrich (Burlington, MA, USA). The ink was placed in an ultrasonic bath for 30 min to reach a well-dispersed mixture. Simultaneously, a glassy carbon disk electrode (GC, 3 mm diameter) was polished to mirror-finish with 0.3 µm and 0.05 µm alumina, washed with distilled water and later sonicated in ethanol for 5 min. Then, 3.5 µL of the carbon ink was cast on the polished glassy carbon disk electrode. Subsequently, the GC electrode with the carbon ink was dried at 70 °C for 24 h in order to obtain a biocarbon (biochar) film employed as a working electrode. The counter electrode was a Pt-wire (Alfa-Aesar, Ward Hill, MA, USA), and an Hg/Hg_2_SO_4_ electrode (E = 0.68 V vs. NHE) was used as a reference. The supporting electrolyte consisted of H_2_SO_4_ 0.5 M. Subsequently, cyclic voltammetry (CV) was carried out in a range from −0.32 to 1.38 V vs. NHE at a scan rate of 50 mV s^−1^ in the cathodic direction in order to identify the electrochemical profile of the obtained carbon materials. Afterward, the electrochemical stability of the carbon materials was evaluated by cyclic voltammetry by first cycling the carbon materials from −0.32 V to 2.18 V vs. NHE in the cathodic direction at a scan rate of 50 mV s^−1^ in acidic media for 20 cycles; then, CV experiments were performed at the initial conditions, from −0.32 to 1.38 V vs. NHE at 50 mV s^−1^, in order to compare the electrochemical behavior of the carbon materials before and after an electrochemical oxidation influence. Additionally, cyclic voltammetry was performed within the non-faradic region of the resulting carbon materials at different scan rates, (10, 25, 50, 100, 150 and 250 mV/s) to determine the double-layer capacitance as well as for the oxidized materials. The described methodology was employed for all the carbon materials obtained from nopal, mango seed and coffee grounds under N_2_ or H_2_ atmospheres at 600 °C, 750 °C and 900 °C and the electrochemical experiments were performed three times to ensure reproducibility. In the following section, the potential E is reported in respect to the normal hydrogen electrode (NHE) for easy comparison.

## 3. Results and Discussion

**Figure 3:**
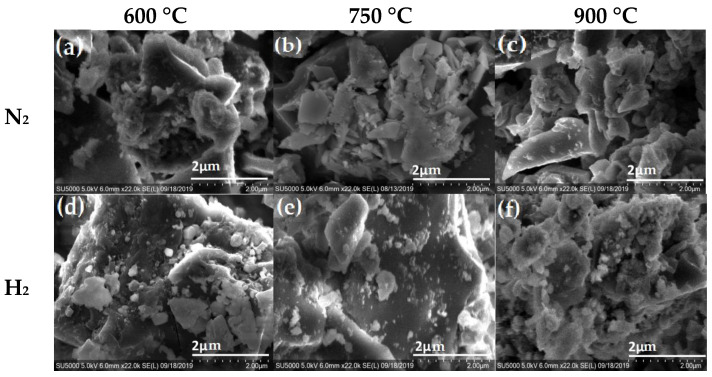
SEM micrographs of pyrolyzed nopal in N_2_ atmosphere (**top**) at (**a**) 600°C, (**b**) 750°C (**c**) 900 °C and H_2_ atmosphere (**bottom**) at (**d**) 600 °C, (**e**) 750 °C and (**f**) 900 °C.

**Figure 4:**
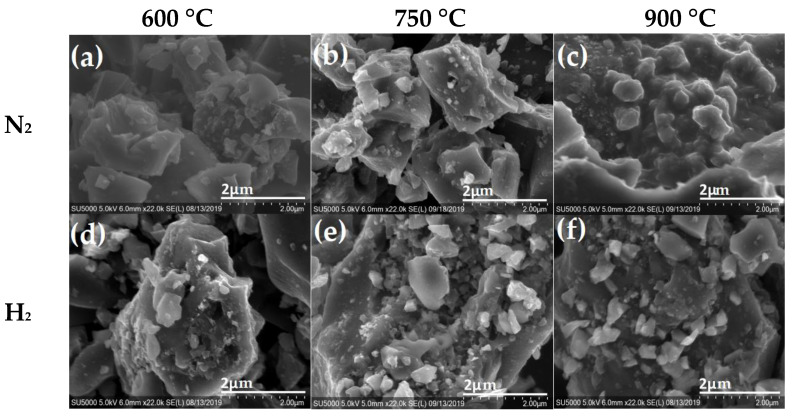
SEM microscopy of pyrolyzed coffee in N_2_ atmosphere (**top**) at (**a**) 600°C, (**b**) 750°C (**c**) 900 °C and H_2_ atmosphere (**bottom**) at (**d**) 600 °C, (**e**) 750 °C and (**f**) 900 °C.

**Figure 5:**
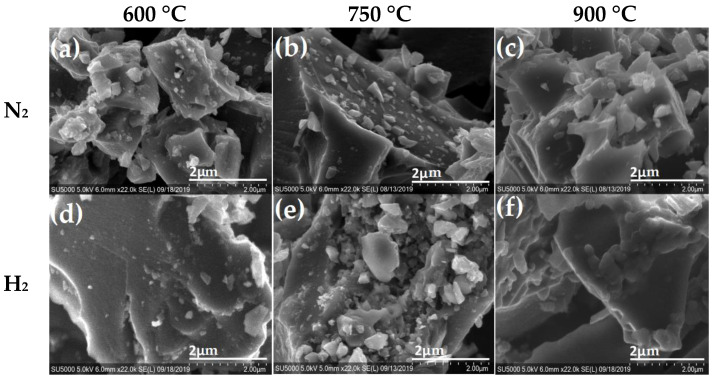
SEM microscopy of pyrolyzed mango in in N_2_ atmosphere (**top**) at (**a**) 600 °C, (**b**) 750 °C (**c**) 900 °C and H_2_ atmosphere (**bottom**) at (**d**) 600 °C, (**e**) 750 °C and (**f**) 900 °C.

The morphological structure of the carbon materials was analyzed using scanning electron microscopy (SEM). This technique is capable of producing high-resolution images that provide information on any change caused at the surface of the carbon material by varying the temperature and atmosphere during the pyrolysis process. SEM images of the carbon materials obtained from nopal, coffee grounds and mango seed are shown in Figure 3, Figure 4 and Figure 5, respectively, under N_2_ and H_2_ atmospheres at 600 °C, 750 °C and 900 °C. Figure 3 shows SEM micrographs for nopal thermally treated with a N_2_ atmosphere (top) and a H_2_ atmosphere (bottom). At 600 °C, it is possible to observe small irregular particles (>1 µM) that agglomerate on the surface; the features of the obtained materials seem to be related to the pyrolysis atmosphere and change with increasing temperature. For instance, it is clear that the N_2_ atmosphere at 900 °C (Figure 3c) favors the formation of carbon materials with a large amount of particles less than 2 μm on the surface as compared with materials obtained at 600 or 750 °C. The samples pyrolyzed in a H_2_ atmosphere at 600 °C also show particles deposited on the surface, (Figure 3d); however, the particles seem to be of a smaller size as compared with those observed for the nopal in the N_2_ atmosphere at 600 °C. Additionally, as the temperature increases, the biocarbon (biochar) from nopal in H_2_ atmosphere seems to reach a smooth surface covered with very small particles ( <<1μm), as shown in Figure 3f.

In Figure 4, the SEM images of coffee pyrolyzed in N_2_ and H_2_ atmospheres are shown. The micrographs of the carbon material obtained under a H_2_ flow exhibit the presence of microscopic particles with cavities on the surface and small particles (>1 µm), deposited on it as the pyrolysis temperature increases from 600 °C to 750 °C, as shown in Figure 4d,e, respectively. At 900 °C, it is observed that some of the small particles on the surface formed large agglomerates with practically an absence of cavities on the surface, shown in Figure 4f. On the other hand, the coffee samples treated with a N_2_ atmosphere exhibit mainly the formation of irregular particles (1 µm approx.), on top of the surface of larger particles in comparison with those samples treated under H_2_ atmosphere at 600 °C and 700 °C. At higher temperatures, it can be observed that the small particles were agglomerated and encrusted on the surface, thus obtaining decorated rough particles, as seen in Figure 4c.

Figure 5 shows that the morphology of mango samples treated with atmospheres of N_2_ and H_2_ revealed some differences to those exhibited by the nopal (Figure 3) and coffee samples (Figure 4). Firstly, large microscopic particles (>>2 µm), are observed for the biomass pyrolyzed under either N_2_ or H_2_ atmosphere. Under N_2_ atmosphere, it seems that the surfaces of these large particles are cleaner as compared to that obtained for nopal and coffee wastes under N_2_ atmosphere since the presence of small particles (>1 µm) is limited. Nonetheless, the pyrolysis of mango under H_2_ atmosphere demonstrated a surface decorated with particles smaller than 1 µm at a temperature of 750 °C (Figure 5e) and 900 °C (Figure 5f). It is important to note that the accumulation of globular particles on the surface of mango is highly evident at 900 °C under the effect of H_2_ atmosphere.

**Figure 6:**
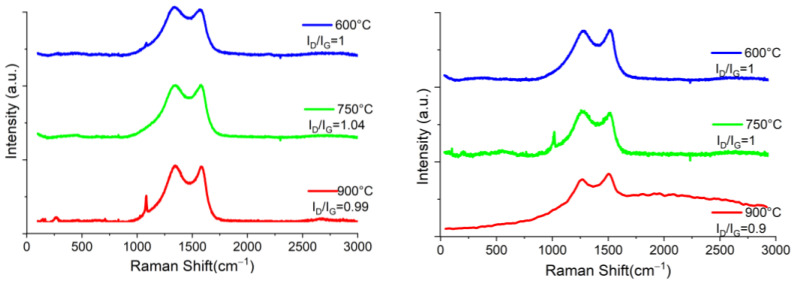
Raman spectra of pyrolyzed nopal in N_2_ atmosphere (left column) and H_2_ atmosphere (right column) at 600 °C, 750 °C and 900 °C, from top to bottom.

**Figure 7:**
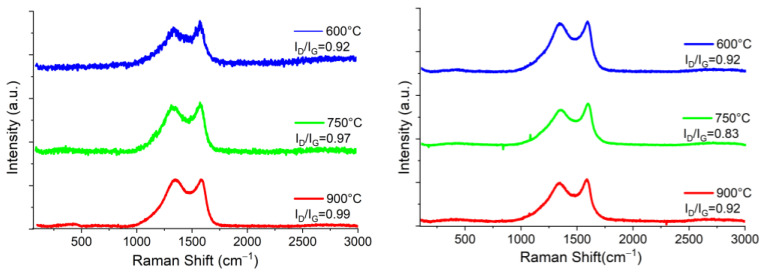
Raman spectra of pyrolyzed coffee in N_2_ atmosphere (left column) and H_2_ atmosphere (right column) at 600 °C, 750 °C and 900 °C, from top to bottom.

**Figure 8:**
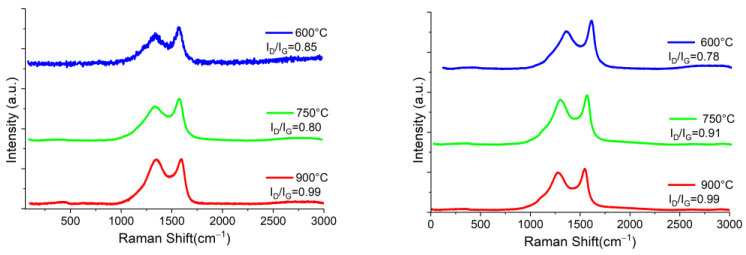
Raman spectra of pyrolyzed mango in N_2_ atmosphere (left column) and H_2_ atmosphere (right column) at 600 °C, 750 °C and 900 °C, from top to bottom.

Raman spectroscopy studies of biochars from nopal, coffee grounds and mango seed are reported in Figure 6, Figure 7 and Figure 8. Overall, the Raman spectra of the prepared biochars reveal the characteristic bands of carbonaceous structures called G band (~1575 cm^−1^) and D band (~1350 cm^−1^). The D band is commonly associated with a mode of respiration of sp^2^ carbon rings, and offers information on structural defects; it is also important to mention that the larger the D band, the higher the degree of defects present in the studied material [28]. These may be punctual, linear, superficial, and volume defects. The most common include voids, cracks, and dislocations, as well as optics that are commonly used in optoelectronics. Furthermore, the broadening of the D band predicts that the samples come from highly disordered materials. The G band located at ~1575 cm^−1^ indicates the graphitic order of the material and is associated with the relative movement of atom pairs of carbon s joined by sp^2^ bonds along the direction of the bond. This band has the characteristic of growing larger and decreasing in intensity until it disappears for amorphous materials. In addition, it is possible to identify a small weak band, about 2650 cm^−1^, called G’, which corresponds to an overtone (second order) of the G band. This can be produced due to a non-uniform structure and therefore, there are different bond energies. To reveal the existence of nested peaks, a deconvolution analysis by a Lorentzian adjustment was carried out. The analysis revealed that overlaid peaks are not present for all Raman spectra obtained, indicating only a possible laser saturation during the measurement, or the existence of a high disorder. Likewise, the relationship between the intensities of the D and G bands (I_D_/I_G_) provided information on the extent of the disorder since this ratio predicts the amount of defects or crystalline limits of the carbonaceous material [29]. If this ratio is close to 1, the carbon material is highly disordered and a less graphitic structure is obtained; otherwise, a graphitic material will have a ratio close to zero.

From the spectra, it is possible to see that the intensities and locations of the bands are similar for all samples regardless of the pyrolysis atmosphere, except for the mango samples, whose intensities are relatively higher than the other biochars. This could mean that the physical characteristics of biochars depend mainly on the organic starting material, since the temperature and atmosphere of pyrolysis could influence the degree of graphitization and disorder. Likewise, the I_D_/I_G_ ratio is related to the density of the defects of graphite, and in our case could also indicate the formation of graphene oxide [30].

Furthermore, the I_D_/I_G_ ratio calculated for carbonaceous materials is inversely proportional to the size of the crystallites in the parallel direction to the basal planes of the graphitic material, as reported by Ferrari-Robertson, Equation (3), [29]:

(3) $$L_{a}=\left(-12.6+0.033\lambda \right){\left(\frac{I_{D}}{I_{G}}\right)}^{-1}$$

where L_a_ is the average size of the grain or crystallite in nm and λ is the wavelength of the equipment used for the measurement; for this study, λ = 532 nm.

The size of the crystallite provides information regarding the predominant domains in the graphite structure. For example, a high value of L_a_ shows a higher content of sp^2^ domains, which is related to the formation of hexagonal graphite or mainly rhombohedral graphite structures [31]. This means that these biochars not only have a graphitic profile, but also that pyrolysis promoted the formation of hexagonal graphite; hence, these hexagonal arrangements favored π bonds that allowed a delocalization of electrons converting the graphite into an electrically conductive material, a desirable property in our work [32]. The value of L_a_ for each carbon material prepared in this work via pyrolysis in H_2_ and N_2_ atmospheres at different temperatures are reported in Table 1.

According to Table 1, it is possible to see that the average size of the crystallite is quite similar for all the samples. The lower value of L_a_ was obtained for nopal at 600 °C in N_2_ atmosphere, at 4.77 nm, and the higher value for mango at 600 °C, which was 6.34 nm, in H_2_ atmosphere, although mango also exhibited a similar L_a_ in N_2_ atmosphere at 750 °C, at 6.20 nm. However, it is clear that the pyrolysis temperature does not define the size of the crystallite among the prepared materials and, also, it seems that pyrolysis atmosphere does not have an influence on it.

Electrical conductivity is an important property for any electrocatalyst support since it must directly facilitate the transport of electrons toward electrochemical reactions. Despite the increase in the pyrolysis temperature, the pressure applied on the sample has a strong effect since, by compressing the sample, the inter-distance between particles is decreased. Thus, the free volume in the sample is reduced and the number of electrical contacts is increased, resulting in the enhancement of electron transport [33]. Particularly, the biochar obtained from nopal, coffee and mango demonstrated that their electrical conductivity increased linearly with the applied pressure, irrespective of the pyrolysis temperature and atmosphere. Nonetheless, it was not possible to reach a maximum value of electrical conductivity within the applied pressure range as expected for the typical conductivity behavior of a packed powder through its percolation threshold and shift from insulator to electrical conductor material: the so-called S curve [20]. Although the highest pressure applied during the conductivity experiments was considered to be enough to compact the sample volume to the minimum, it was not possible to fully obtain the conductivity spectra of the biochar as a function of pressure.

**Figure 9:**
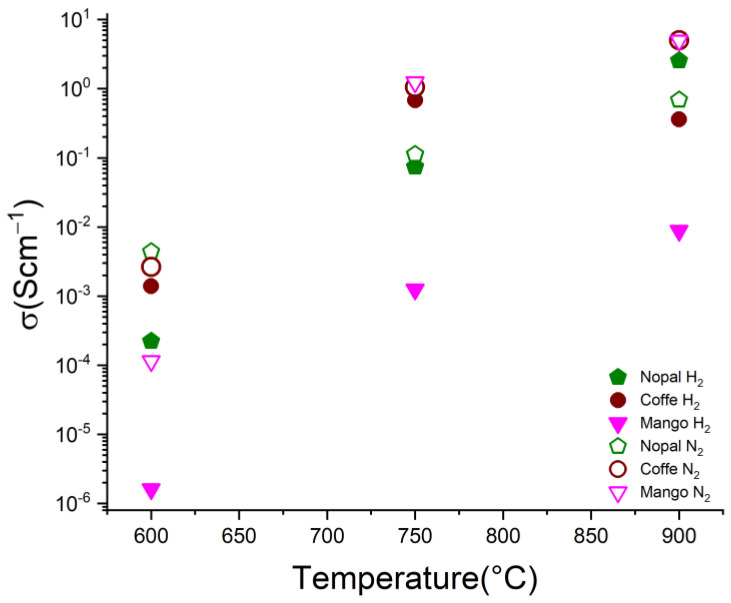
Electrical conductivity of biochars from nopal, coffee grounds and mango as a function of the pyrolysis temperature at an applied pressure of 620 kPa.

In Figure 9, the electrical conductivity values of the biochars at the highest applied pressure (620 kPa) are reported for different pyrolysis temperatures and atmospheres. According to these results, it is possible to see that the electrical conductivity of the biochars clearly increased as the pyrolysis temperature increased, regardless of the pyrolysis atmosphere. Furthermore, the prepared biochar demonstrated a conductivity in the range of 10^−4^ to 10^1^ S cm^−1^. In particular, the coffee sample pyrolyzed with a N_2_ flow at 900 °C and the mango biochar at 900 °C in N_2_ and H_2_ atmospheres exhibited the highest electrical conductivity values of ~10^1^ S cm^−1^. These results fall within the electrical conductivity values reported in the literature for a number of lignocellulosic-derived biochars, i.e., from 10^−6^ to 10^2^ S cm^−1^ [26,34,35]. For instance, a corncob biochar and light-roast coffee chaff produced by pyrolysis at 900 °C in a N_2_ atmosphere demonstrated lower conductivities of ~15 mS cm^−1^ and 3.5 mS cm^−1^, respectively, under similar pressures to those reported in this study [26,36]. Therefore, the increase in the electrical conductivity of the biochars as a function of the temperature is associated with an enhancement of graphitic-phase crystallinity in the samples, as well as with the formation of smaller particle sizes (<2 μm in average) as the pyrolysis temperature was increased (please see SEM results [24]). These factors could improve the flow of electric current for our biochars at 900 °C, especially for mango and coffee biochars as reported above. Moreover, the obtained electrical conductivity values would be associated to the type of the carbonaceous materials obtained in this work, such as their chemical composition, i.e., O, N, C, H or any metal content, as well as lignin content, surface area, conductive graphite content or size of graphitic crystallites, among other factors influencing electrical conductivity [34,37]. Further research in this area is being considered in order to gain a better understanding of the conductivity behaviors of these biochars.

The specific surface area (SSA) of the prepared biochar from nopal, coffee grounds and mango is reported in Table 2. It is noted that there is not a clear correlation present in all biochars between SSA value and pyrolysis temperature increase. For instance, nopal and mango in both N_2_ and H_2_ flows demonstrated that the specific surface area decreased as the pyrolysis temperature increased; while coffee in N_2_ exhibited the opposite behavior, SSA increased as the pyrolysis temperature increased. Coffee in H_2_ did not show a specific trend exhibiting the maximum SSA at 750 °C. Regarding SEM results, as previously mentioned, the decrease in the SSA as the pyrolysis temperature increased was associated with the formation of particle agglomerates and also with smoother surface particles in the biochars at higher temperatures. Thus, it is assumed that the adsorption of inert molecules, such as nitrogen and hydrogen, greatly depends on the topology and not at all on the chemical nature of the surface material [38]. Among all the biochars, mango in H_2_ atmosphere (829 m^2^/g) and nopal in N_2_ atmosphere (766.7 m^2^/g) at 600 °C demonstrated the highest specific surface area. Nonetheless, coffee in H_2_ at 750 °C (699.3 m^2^/g), nopal in H_2_ at 600 °C (568.2 m^2^/g) and nopal in H_2_ at 750 °C (550.6 m^2^/g) also showed attractive SSA values suitable for electrocatalyst supports. The specific surface areas of the biochars reported in this work are in good agreement with those reported for similar materials in the literature [2], which demonstrated specific areas between 569 and 328 m^2^/g. Additionally, Vulcan XC-72, a widely used carbon black as an electrocatalyst support, presents an SSA of 218 m^2^/g [39]. Note that this value is remarkably lower than the SSA results reported in Table 2. Therefore, the biochars reported in this work could be considered, at first sight, as an alternative option to Vulcan XC-72 as an electrocatalyst support for electrochemical reactions of interest in energy production systems.

**Figure 10:**
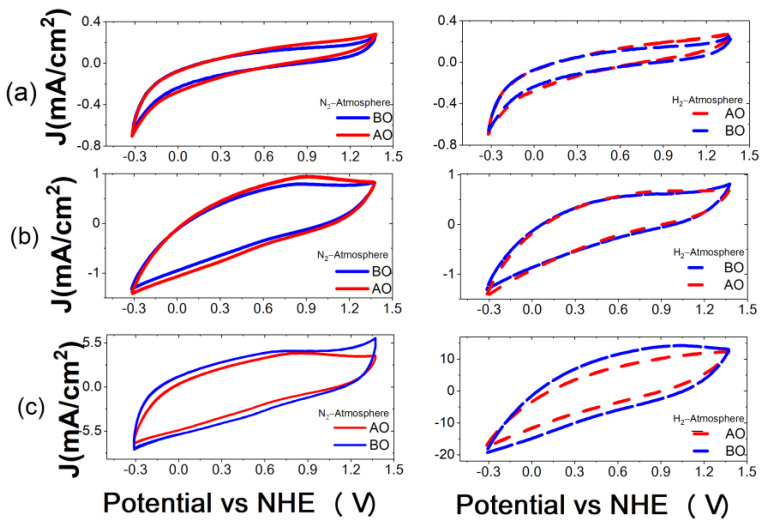
Cyclic voltammograms of biochars from nopal at (**a**) 600 °C, (**b**) 750 °C and (**c**) 900 °C in N_2_ atmosphere (solid lines) and H_2_ atmosphere (dashed lines) before (BO, blue) and after (AO, red) electrochemical oxidation. Electrolyte solution: H_2_SO_4_ 0.5 M. Scan rate: 50 mV/s.

**Figure 11:**
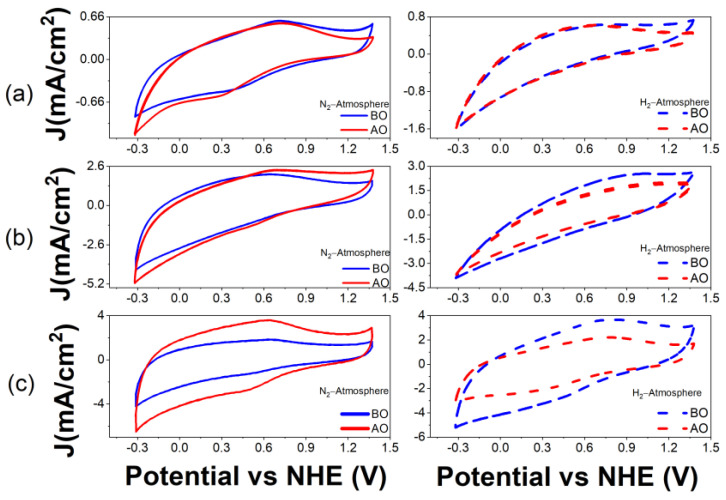
Cyclic voltammograms of biochars from coffee at (**a**) 600 °C, (**b**) 750 °C and (**c**) 900 °C in N_2_ atmosphere (solid lines) and H_2_ atmosphere (dashed lines) before (BO, blue) and after (AO, red) electrochemical oxidation. Electrolyte solution: H_2_SO_4_ 0.5 M. Scan rate: 50 mV/s.

**Figure 12:**
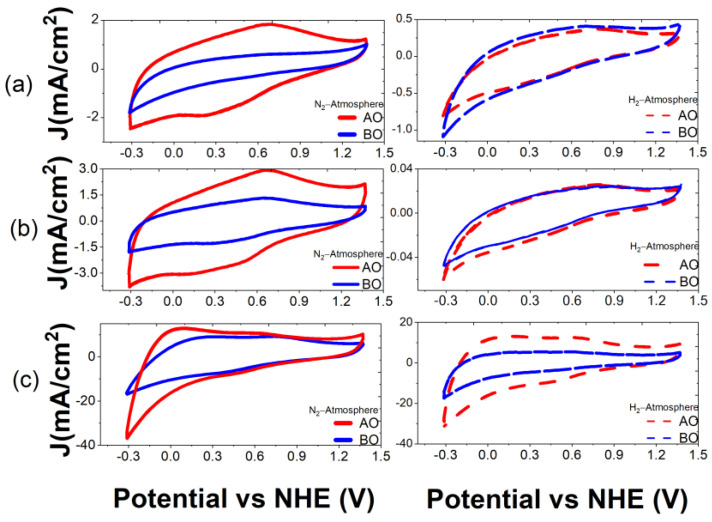
Cyclic voltammograms of biochars from mango at (**a**) 600 °C, (**b**) 750 °C and (**c**) 900 °C in N_2_ atmosphere (solid lines) and H_2_ atmosphere (dashed lines) before (BO, blue) and after (AO, red) electrochemical oxidation. Electrolyte solution: H_2_SO_4_ 0.5 M. Scan rate: 50 mV/s.

In addition to the physicochemical characterizations described above, an electrochemical study was carried out for each of the biochars in order to evaluate their behavior in acidic media, their electrochemical stability and their double-layer capacitance. The electrochemical stability of each of the biochars was evaluated by comparing the CVs in a window of −0.32 to 1.38 V vs. NHE at a scan rate of 50 mV/s before and after oxidation, as described in Section 2.3 and as reported in Figure 10, Figure 11 and Figure 12.

The cyclic voltammograms of the nopal samples do not show the oxidation and reduction peaks related to the hydroquinone/quinone redox reactions, which is typically observed in the region of 0.4–0.8 V vs. NHE by CV for carbonaceous materials [40]. Thus, electrochemical reactions are not observed for this biochar. Additionally, the nopal biochar exhibits good electrochemical stability since there are no considerable changes in the electrochemical behavior of the CV before and after the oxidation process. This is because the current density is quite similar regardless of the temperature and atmosphere during the pyrolysis process.

Figure 11 and Figure 12 indicate that the same biochars can have different electrochemical behaviors depending on the atmosphere or pyrolysis temperature. Coffee and mango biochars pyrolyzed in a N_2_ atmosphere demonstrated electrochemical reduction and oxidation peaks in contrast to materials pyrolyzed in H_2_ atmosphere, since no defined peaks were observed and thus, no electrochemical reactions occurred at the surface of these biochars, such as at 600 and 700 °C. The samples pyrolyzed in a N_2_ atmosphere showed oxidation peaks between 0.65 V and 0.71 V vs. NHE, which were associated with the quinone groups; however, the potential of the reduction peaks shift, depending on the biochar, at different pyrolysis temperatures. For the biomass pyrolyzed at 600 °C, a reduction peak is located at approximately 0.33 V, and for the biomass pyrolyzed at 900 °C, at 0.51 V. These peaks are usually attributed to the reduction in hydroquinone groups on the surface [41]. No reduction peak is observed in the sample pyrolyzed at 750 °C. In addition, it is possible to see that the samples show a higher current after oxidation, in particular the one pyrolyzed in N_2_ atmosphere at 900 °C. This means that oxygen groups were generated on the surface of the biochar during the electrochemical oxidation process by CV.

After the oxidation process, the mango biochars in N_2_ atmosphere at 600 °C and 750 °C showed oxidation and reduction peaks at approximately 0.23 V and 0.67 V, respectively, corresponding to the redox behavior of the hydroquinone and quinone groups. Additionally, the redox process related to the quinone/hydroquinone is not evident at all for the mango biochar obtained at 900 °C in a N_2_ flow. However, it seems that this redox process originated after electrochemical oxidation (0.3–0.7 V), but overlapped with an additional electrochemical process, since the density current was enhanced at a negative potential, −0.3 V. This phenomenon could be attributed to an electrochemical activation process of the mango biochar, thus exhibiting an electrocatalytic behavior toward the hydrogen reduction reaction. A similar behavior was raised for the mango pyrolyzed in H_2_ atmosphere at 900 °C, indicating that similar carbon structures could have been obtained regardless of the pyrolysis atmosphere. Finally, the cyclic voltammograms of some of the biochars in this work exhibited a similar electrochemical behavior to Vulcan XC-72 [42,43], such as the mango biochar in N_2_ at 600 °C and 750 °C and coffee in N_2_ at 900 °C.

Once the cyclic voltammograms (CVs) of the biochars were obtained at a potential window of −0.30 to 1.37 V vs. NHE, a non-faradaic region was selected for each of them and cyclic voltammetry was carried out at different scan rates of 10, 25, 50, 100, 150 and 250 mV/s in order to determine the double-layer capacitance of the carbonaceous materials prior to any electrochemical oxidation, as described in Section 2.3. Subsequently, the biochars were subjected to cyclic voltammetry measurements at a wider potential window (−0.30 to 1.87 V vs. NHE) in order to promote the electrochemical oxidation of the biochar carbon structure [40,44]; likewise, CVs in a non-faradaic region were performed at different scan rates, as described above, after the oxidation experiments in order to evaluate the capacitance, of the now oxidized biochars. Here, it is important to recall that the capacitance is an indirect method to evaluate the electrochemical active area (ECA), as well as the movement of ions at the electrode interface. Moreover, the capacitance of the biochars was estimated to evaluate their electrochemical stability in acidic media, since carbon materials within an energy storage system are subjected to anodic/cathodic potentials cycles, and carbon corrosion could occur. It is reported that carbon corrosion contributes to an accelerated loss of the active surface area, as well as to the electrical conductivity. This compromises electron transport between the support and the surface of the electrocatalyst. The kinetic performance of any electrochemical reaction is thus decreased [45]. Consequently, carbon supports with high electrochemical stability are of great interest for designing high-performance energy production systems, and so, the biochar capacitance was estimated using the equation:

(4) $$J=\frac{C}{v}$$

where C is the capacitance in F/g, J is the current density in A/g, and v is the scan rate in V/s.

From Equation (4), a graph of J vs. v^−1^ was constructed from the current density data acquired from the CVs at different scan rates in a non-faradaic region by performing a linear fitting, and it was possible to determine the slope, which was directly related to the capacitance of the biochars. Thus, the capacitance values of the biochars were considered to estimate their electrochemical active area (ECA), as well as to evaluate the electrochemical stability of the prepared biochars, as shown in Table 3.

**Figure 13:**
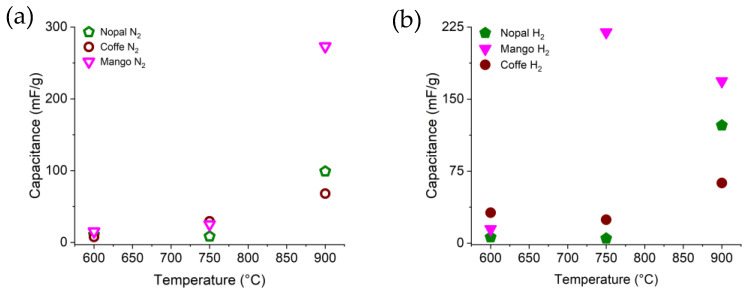
Relation of the capacitance of pyrolyzed biochar (nopal, coffee and mango) prior to oxidation as a function of the pyrolysis atmosphere in (**a**) N_2_ and (**b**) H_2_.

In Table 3, it is observed that the capacitance of the biochars was highly random among of all them prior to and after oxidation. However, the estimated capacitance of some of the biochars are in good agreement with those reported by other authors in the literature (200 mF/g) [46,47]. The mango biochar demonstrated the highest capacitance values among all the biochars in H_2_ at 750 °C (219.55 mF/g) and N_2_ at 900 °C (273.08 mF/g), before oxidation, as seen in Table 3 and Figure 13. Furthermore, Table 3 shows that the mango biochar in H_2_ at 900 °C increased its capacitance from 168.58 mF/g to 296.02 mF/g after oxidation, demonstrating good electrochemical stability. In contrast, coffee biochar in H_2_ showed a decrease in capacitance and therefore, poor electrochemical stability. This may be due to the chemical and physical properties achieved at the considered pyrolysis conditions, such as the greater number of I_D_/I_G_ defects reported for the mango biochar that facilitate the surface hydrophilicity of the carbon structure due to the formation of functional groups containing oxygen [48], thereby increasing the area accessible to electrons. According to Husain et al. [49], this hydrophilicity is also produced by the increased pyrolysis temperature. Finally, the mango biochar at 900 °C also demonstrated the highest specific surface area and electrical conductivity in this work, which would also explain such high capacitance values with respect to the other pyrolyzed materials. Here, a further analysis of the chemical composition of these biochars is considered in order to gain better insight into the enhancement of both physicochemical and electrochemical properties regarding the O, N, C and H percentages, or any metal content, particularly for the mango biochar.

Figure 13 shows graphs of C vs. T in order to evaluate the relationship between the capacitance of the biochars as a function of the pyrolysis temperature under N_2_ and H_2_ atmospheres. Overall, it is possible to observe that the capacitance of the biochar could be related to the pyrolysis temperature, regardless of the atmosphere, since it increased as the temperature increased. Biochars under pyrolysis treatment at 900 °C are then expected to demonstrate a higher capacitance. Note that the mango biochar at 750 °C in H_2_ exhibited a larger capacitance than the same biochar in N_2_. Nonetheless, mango in N_2_ at 900 °C demonstrated a higher capacitance than mango in H_2_ at 750 °C. Furthermore, it is also observed that the pyrolysis atmosphere had an impact on this property since biochars in N_2_ apparently reported a higher capacitance, especially mango in N_2_ at 900 °C.

## 4. Conclusions

Biochar materials made from nopal, coffee grounds and mango residues by pyrolysis under N_2_ and H_2_ atmospheres at temperatures of 650 °C, 700 °C and 900 °C were investigated to obtain insight into their chemical, physical and electrochemical properties regarding their further use as electrocatalyst supports. The results showed that both atmospheres, N_2_ and H_2_, allow the graphitization of organic residues. In addition, it was possible to observe that electrical conductivity and capacitance increased as a function of the pyrolysis temperature, while specific surface area decreased for some biochars as the pyrolysis temperature raised. The effects of the N_2_ and H_2_ atmospheres during the pyrolysis on these parameters were mostly irrespective since similar results were obtained; nonetheless, biochars in N_2_ seem to present a higher electrochemical stability than those obtained in a H_2_ atmosphere. Furthermore, mango residues pyrolyzed at 900 °C in a N_2_ atmosphere demonstrated the best properties to be considered as a suitable alternative carbon material for electrocatalyst support, since they exhibited high electrical conductivity, good capacitance and a specific surface area similar to Vulcan XC-72, as well as high electrochemical stability in acidic media. Finally, this study demonstrated that it is possible to add value to different organic waste in a straightforward way, without the need for complicated synthesis methods, sophisticated equipment and/or expensive reagents.

## Figures and Tables

**Table 1:** The crystallite size (L_a_) values for the biochar obtained in N_2_and H_2_ atmospheres at different temperatures.

L_a_ (nm)						
Sample	600 °C		750 °C		900 °C	
	N_2_	H_2_	N_2_	H_2_	N_2_	H_2_
Mango	5.83	6.34	6.20	5.44	5.07	5.07
Coffee	5.39	5.38	5.11	5.97	5.09	5.38
Nopal	4.77	4.96	5.00	4.96	4.96	5.51

**Table 2:** Specific surface area (SSA) of biochars (nopal, coffee and mango) at 600, 750 and 900 °C in atmospheres of N_2_ and H_2_.

SSA (m^2^/g)						
Sample	600 °C		750 °C		900 °C	
	N_2_	H_2_	N_2_	H_2_	N_2_	H_2_
Nopal	766.7	568.2	263.5	550.6	179.3	462. 6
Coffee	171.0	126.0	231.0	699.3	276. 5	225.3
Mango	334.2	829.0	143.0	305.0	140.0	289. 1

**Table 3:** Capacitance of biochars (nopal, coffee and mango) at 600, 750 and 900 °C in atmospheres of N_2_ and H_2_.

Capacitance (mF/g)						
Sample	600 °C		750 °C		900 °C	
	BO	AO	BO	AO	BO	AO
Coffee H_2_	31.68	11.54	24.39	10.56	62.63	35.25
Mango H_2_	14.56	10.56	219.55	7.28	168.58	296.02
Nopal H_2_	6.19	4.73	4.95	5.24	122.70	160.93
Coffee N_2_	7.65	10.92	29.49	16.75	68.09	152.56
Mango N_2_	15.29	9.47	25.12	48.06	273.08	158.02
Nopal N_2_	10.92	13.84	8.37	5.10	99.04	61.53

BO = Before oxidation. AO = After oxidation.

## Data Availability

Not applicable.
